# Community health professionals’ dementia knowledge, attitudes and care approach: a cross-sectional survey in Changsha, China

**DOI:** 10.1186/s12877-018-0821-4

**Published:** 2018-05-25

**Authors:** Yao Wang, Lily Dongxia Xiao, Yang Luo, Shui-Yuan Xiao, Craig Whitehead, Owen Davies

**Affiliations:** 1 Xiang Ya School of Nursing, Central South University, Changsha, Hunan Province China; 2 Xiang Ya School of Public Health, Central South University, Changsha, Hunan Province China; 3 College of Nursing and Health Sciences, Flinders University, Adelaide, South Australia Australia; 4 Flinders Medical Centre and Flinders University, Adelaide, South Australia Australia

**Keywords:** Dementia, Knowledge, Attitudes, Care approach, Community health professionals

## Abstract

**Background:**

Community health professionals play a significant role in dementia care. However, little is known about community health professionals’ capacity in dementia care, especially in low and middle-income countries. The aim of the present study was to assess community health professionals’ dementia knowledge, attitudes and care approach in China, a country with the largest population of people with dementia in the world and where community based dementia care services are much needed.

**Methods:**

A cross-sectional survey was conducted. 450 health professionals were recruited into the study using random sampling from community health service centres in Changsha, China. Their knowledge, attitudes and care approach were assessed utilising the Chinese version of the Alzheimer’s Disease Knowledge Scale, Dementia Care Attitude Scale and Approach to Advanced Dementia Care Questionnaire respectively.

**Results:**

A total of 390 participants returned the questionnaire (response rate 87%). Age, education, professional group and care experience were associated with knowledge scores, and overall dementia knowledge was poor. Attitudes were generally positive and influenced by age, professional group, gender and care experience. The experience of caring for people with dementia was positively associated with a person-centred care approach, although the participants tended not to use a person-centred care approach. A statistically significant association was found between knowledge and attitudes (*r* = 0.379, *P* < 0.001), and between attitudes and care approach (*r* = 0.143, *P* < 0.001). However, dementia knowledge has no relationship with a person-centred approach.

**Conclusions:**

Community health professionals showed generally positive attitudes towards people with dementia. However, they demonstrated poor dementia knowledge and tended not to use a person-centred care approach. The results suggest that a multifaceted approach consisting of educational interventions for community health professionals, and policy and resource development to meet the demand for community dementia care services, is urgently needed in China.

## Background

Dementia has become a priority global health issue in the context of an ageing population. It is estimated that there were around 47 million people worldwide living with dementia in 2016 and this number will increase to 131 million by 2050 [1]. The majority of people with dementia live in low and middle-income countries (LMICs) and are cared for by family members at home [2]. China shows a faster growing ageing population than most other nations in the world. The 2015 World Alzheimer Report revealed that around 9.5 million people with dementia lived in China, comprising 20% of the total number of people in the world with dementia [2]. This disease burden places great pressure on the Chinese healthcare system as well as a level of demand for health professionals to provide competent dementia care greater than exists in other nations [2, 3]. Community health professionals have a significant role to play in dementia care, however, studies on their capability to provide dementia care are limited and mainly come from high-income countries. This study addressed the gap in research by examining the knowledge, attitudes and dementia care approach of community health professionals from one LMIC.

Studies have demonstrated that early detection and diagnosis of dementia enabled people with dementia to receive adequate treatment, supported their family caregivers to manage dementia at home and reduced preventable dementia associated functional decline and complications [4, 5]. Early post-diagnosis care and continuing care were associated with improved quality of life of people with dementia and relieved caregiver burden [1, 6]. Moreover, caring for people with dementia through the provision of community dementia care services was shown to be far less costly than in residential aged care facilities [4, 7]. Providing community dementia care services has become a strategy in the dementia action plan of high-income countries and is highly recommended in the World Health Organization Global Action Plan which also incorporates preparing community health professionals to provide competent dementia care [7].

Dementia care is not yet integrated into the medical and nursing baccalaureate curricula in China [8]. Although community health professionals in China are required to attend continuing professional development programmes to gain re-registration, most programmes rarely include dementia care. Studies have identified self-reported insufficient knowledge to deal with dementia, difficulties in detecting early signs of dementia, dementia diagnosis and post-diagnosis management among community health professionals [6, 8]. Furthermore, current care practice in dementia service was largely task-focused and not delivered in a person-centred way, which lead to an increase in agitated behaviours, falls and additional distress for people with dementia [9, 10]. Studies also revealed health professionals’ inadequate attitudes towards people with dementia and their caregivers [10, 11]. For example, a large-scale survey conducted in England reported that health professionals perceived that ‘managing dementia is more often frustrating than rewarding’ and ‘the primary care team has a limited role to play in the care of people with dementia’ [12].

This knowledge deficit about dementia was one of the most important factors that affected the ability of community health professionals to identify and diagnose people with dementia [13, 14]. Inappropriate and delayed treatment of dementia, and misinterpretation of behavioural and psychological symptoms of dementia (BPSD) were reported and attributed to lack of dementia knowledge [15–17]. Furthermore, negative attitudes towards people with dementia among health professionals were associated with less recognition of dementia [18–20]. On the other hand, health professionals with a higher level of dementia knowledge and positive attitudes were more likely to detect and diagnose dementia in a timely manner and demonstrated a greater tendency to employ a person-centred dementia care approach compared to those with poor dementia knowledge and attitudes [11, 21].

The variables affecting health professionals’ dementia knowledge, attitudes and approaches to dementia care have been studied. In a cross-sectional study with 387 hospital nurses in Taiwan by Lin and colleagues, the socio-demographic characteristics of health professionals were associated with dementia knowledge [22]. In this study, being of an older age, holding a bachelor degree and having an interest in dementia care were positively associated with dementia knowledge. The findings echoed those of another study where older health professionals demonstrated better dementia knowledge scores than their younger counterparts [23] but no relationships between gender and dementia knowledge and attitudes. However other studies identified that advanced education level was a predictor of dementia knowledge [24, 25]. In a large scale cross-sectional study with 1047 nurses and personal care assistants in aged care in Hong Kong, the researchers identified that exposure to dementia training was associated with a positive attitude towards dementia care [26]. This finding supports the study by Lee and colleagues that nurses who received dementia care training demonstrated higher scores in dementia knowledge compared to those without training [22].

Previous studies have identified that the lack of opportunities for health professionals to engage in dementia specific education is a major barrier to achieving timely diagnosis and dementia management in primary care in China [8, 27]. Dementia education is not yet incorporated into the curricula in formal education programmes for health professional students, nor in continuing professional development programmes in China [8, 27]. There are no stand-alone topics nor any integrated learning content in dementia care across topics in the curricula in formal education programmes and dementia is only very briefly introduced as one of many neurological diseases in the curricula [8]. Measuring health professionals’ knowledge, attitudes and care approach to dementia is a vital step in identifying evidence to inform dementia care education and training. However, as most studies on community health professionals were conducted in high-income countries, the findings of these studies may have limited generalizability in China and other LMICs due to social and cultural differences. Moreover, differences in health policies and systems between high-income countries and LMICs might also affect the application of research evidence in education program development to meet the learning needs of community health professionals. Further studies are needed across nations to add research evidence to the international community to meet the care needs of people with dementia and their caregivers in the community setting.

## Methods

### Study design

A cross-sectional survey of community health professionals was conducted to collect self-reported dementia knowledge, attitudes and care approach.

### Sample and setting

The survey was conducted in Changsha, Hunan province, China. Changsha is the capital and the most populous city in Hunan Province with a population density of 647 people per square kilometre and consists of 6 districts, 1 county and 2 county-level cities. Six districts were selected for this study as a representative sample because they have a similar population structure and economic level compared to each other, and they are located reasonably near to each other, enabling data collection in a timely manner. The estimated ratio of health professionals to population is 1.65 per 1000 population in these six districts ranging from 1.3 nurses per 1000 population to 1.7 general practitioners (GPs) per 1000 population as reported by the Health and Family Planning Commission of China [28, 29]. The percentage of people aged over 60 in these six districts is 17.6%, which is similar to the percentage of people aged over 60 in the general population of China (17.3%) [30, 31]., predicting similar numbers of people living with dementia based on the prevalence rate of 6.6% among people aged 60 or over in China [2]. Therefore the provision of caregiving for people with dementia should also be similar given the same culture and traditions in the care of older people in China.

The target population in the present study was health professionals working in community health services centres (CHSCs). Community health professionals are usually GPs, community nurses and allied health professionals. In China, only 23.8% of GPs have a Bachelor Degree that is equal to Level 6 of the European Qualifications Framework [32, 33]. The majority of GPs and community nurses hold an Associate Degree that is equivalent to Level 5 of the European Qualifications Framework [32]. Since no up-to-date list of community health professionals was available to the researchers, a three-step sampling approach was used for the purpose of this study in order to minimize selection bias and ensure generalizability. First, the CHSCs were stratified into six districts. Second, one CHSC from each district was selected randomly using a random number generator. Third, all of the health professionals employed by the selected CHSCs were invited to participate in the present study.

### Measurements

#### Demographic data

A demographic questionnaire was developed to collect participant information for items related to gender, age, marital status, education, work experience, professional group, and experience in caring for people with dementia.

#### Alzheimer’s disease knowledge scale (ADKS)

The Alzheimer’s Disease Knowledge Scale (ADKS) was used to assess health professionals’ knowledge about Alzheimer’s disease. The ADKS is a single factor scale and contains 30 true/false items covering the following 7 content domains: life impact (3 items), assessment and diagnosis (4 items), symptoms (4 items), disease progression (4 items), treatment and management (4 items), caregiving (5 items), risk factors (6 items) [23]. A total score is calculated by summing the correct scores for each item, yielding a total score ranging from 0 to 30. A higher total score indicates better knowledge.

#### Dementia care attitude scale (DCAS)

The Dementia Care Attitude Scale (DCAS) was used to assess health professionals’ attitudes towards individuals with dementia and their caregivers. The Chinese version of the DCAS contains 8 items with responses scored on a 5-point Likert scale ranging from 1 (strongly disagree) to 5 (strongly agree). Four of eight items are negatively worded and are reversed in a definite order (e.g. a score of 5 becomes 1) when calculating the total score. The total score range is from 8 to 40 with higher scores indicating more positive attitudes.

#### Approach to advanced dementia care questionnaire (ADCQ)

The Approach to Advanced Dementia Care Questionnaire (ADCQ) was used to assess participants’ care approach for people with dementia. The scale assumes a background scenario of a woman with severe dementia who exhibited difficult behaviours. It contains 13 items and measures the care approach from the following five aspects: orientation of time, place and situation; correction of behaviour; emphasis on the past or the present; aim of the communication; and whether confusion had any meaning for the people with dementia [34]. The choice of answers indicates either a person-centred approach (1 point) or a reality-oriented approach (0 points). The total score range is 0–13, with a higher score indicating a greater tendency to employ a person-centred approach.

### Statistical analysis

The data were checked for errors before double-entry computer input. SPSS (Version 22.0) software (IBM Corp., Armonk, New York, USA) was used for data analysis. Descriptive statistics including mean and standard deviations were used to describe demographic data and the ADKS, DCAS and ADCQ scores. The Kolmogorov-Smirnov test was used to assess normality of distribution of all variables. The ADKS, DCAS and ADCQ scores showed normality of distribution. The independent-sample t test and Pearson correlation analysis were used to measure the significance of the ADKS, DCAS and ADCQ scores between groups according to demographic characteristics on the confirmation of the normal distribution of the scores. Multivariate analysis of variance (MANOVA) was used to compare the total score and scores of each content domain of the ADKS and ADCQ between GPs and community nurses. Bonferroni correction was used to adjust the alpha level that was used to judge statistical significance. A correlation analysis was performed to quantify the strength of association between the ADKS, DCAS and ADCQ scores. Variance inflation factor (VIF) was used to check for multi-collinearity prior to multivariate regression analysis [35]. Multivariate regression with “enter” selection procedure was further employed to explore the net effect of independent variables on the ADKS, DCAS and ADCQ scores (dependent variable). Statistical significance was based on *p*-value< 0.05 in 2-tailed tests.

### Ethical considerations

Ethical approval was granted from the Human Research Ethics Committee of Xiang Ya School of Nursing, Central South University (Project Number 20137801). Permission to use the ADKS, DCAS and ADCQ was obtained from the authors who developed them. A covering letter and the questionnaire were distributed to explain the aim and process of the study. Participation in the present study was voluntary and confidential. Returning the questionnaire was a voluntary way to opt-into the study, so no consent form was required [36]. In order to maintain anonymity, health professionals were asked to leave the completed questionnaire in a box file left at the reception area of their work place. All data collected were treated anonymously and confidentially.

## Results

### Participants’ characteristics

Of the 450 questionnaires administered, 390 questionnaires were returned (response rate (87%). The majority of respondents were female (78.7%) and 54.4% were GPs. Health professionals were aged between 18 and 73 years (mean = 31.6 years, SD = 9.6), and more than half of them (52.3%) held a diploma degree. Just over half of the health professionals (50.3%) reported that they had experience in caring for people with dementia. The demographic and professional characteristics of the participants are summarized in Table 1.

**Table 1:** Demographic characteristics and their relationship to the ADKS, DCAS and ADCQ (*n* = 390)

Characteristics	Summary statistics	ADKS Mean ± SD	*t* value or *r* value	*p*-value	DCAS Mean ± SD	*t* value or *r* value	*p*-value	ADCQ Mean ± SD	*t* value or *r* value	*p*-value
Age (years;mean ± SD,range)	31.6 ± 9.6, 18–73	__	0.209	0.001*^b^	__	0.102	0.045*^b^	__	−0.014	0.782 ^b^
Gender, n(%)			1.404	0.161 ^a^		2.166	0.031* ^a^		−0.483	0.630 ^a^
Male	83(21.3)	19.3(2.86)			27.8(3.30)			8.0(2.37)		
Female	307(78.7)	19.8(3.12)			28.7(3.45)			7.8(2.11)		
Marital Status, n(%)			−1.748	0.081 ^a^		−0.676	0.499 ^a^		0.591	0.555 ^a^
Married	226(57.9)	20.0(3.02)			28.6(3.49)			7.7(2.22)		
Other	164(42.1)	19.4(3.12)			28.3(3.35)			7.9(2.09)		
Education, n(%)			2.324	0.021* ^a^		0.364	0.348 ^a^		0.333	0.740 ^a^
Diploma	204(52.3)	19.4(3.05)			28.3(3.46)			7.8(2.15)		
Bachelor and above	186(47.7)	20.1(3.06)			28.7(3.40)			7.8(2.19)		
Professional Group, n(%)			−4.202	0.000* ^a^		−3.188	0.002* ^a^		−1.262	0.208 ^a^
GPs	212(54.4)	20.3(3.01)			29.0(3.56)			7.9(2.28)		
Community nurses	178(45.6)	19.0(3.01)			27.9(3.20)			7.6(2.02)		
Experience in caring for people with dementia, n(%)			−2.348	0.019* ^a^		−2.046	0.041* ^a^		−2.315	0.021* ^a^
Yes	196(50.3)	20.1(3.29)			28.8(3.51)			8.0(2.14)		
No	194(49.7)	19.4(2.79)			28.1(3.32)			7.5(2.17)		

^a^Statistic was based on independent-samples *t* test

^b^Statistic was based on Pearson correlation analysis

*p < 0.05; SD, standard deviation

*ADKS* Alzheimer’s Disease Knowledge Scale, *DCAS* Dementia Care Attitudes Scale, *ADCQ* Approach to Advanced Dementia Care Questionnaire

### ADKS scores

The overall mean score of dementia knowledge was 19.7 (SD = 3.07) out of 30 and equivalent to 66% of correct answers (Table 1). Items with the poorest responses included those related to symptoms and care-giving (percent correct = 42%). A majority of the participants (62.8% *n* = 245) mistakenly thought that tremor or shaking of the hands or arms was a common symptom in people with Alzheimer’s disease. Only 19% (*n* = 74) of health professionals responded correctly to the statement that ‘When people with Alzheimer’s disease repeat the same question or story several times, it is helpful to remind them that they are repeating themselves’. Results also indicated only a 67% correct response rate about dementia risk factors. Only 49% (*n* = 191) responded correctly that ‘people in their 30s can have Alzheimer’s disease’. Most participants (94.1% *n* = 367) responded correctly that ‘People whose Alzheimer’s disease is not yet severe can benefit from psychotherapy for depression and anxiety’. 51% of participants (*n* = 199) responded incorrectly question to the statement that ‘once people have Alzheimer’s disease, they are no longer capable of making informed decisions about their own care’. Results also revealed that GPs demonstrated a higher mean score in risk factors, symptoms and life impact compared to nurses (Table 2).

**Table 2:** ADKS content domains and professional group (n = 390)

				Professionals				
				GPs(*n* = 212)	Community nurses (*n* = 178)			
Content Domain	#items	Mean ± SD	%Correct	Mean ± SD	Mean ± SD	*F* value	*p*-value	ES
ADKS	30	19.7(3.07)	66%	20.3(3.01)	19.0(3.01)	17.653	0.000*	0.211
Risk Factor	6	4.0(1.01)	67%	4.2(0.99)	3.9(1.01)	9.595	0.002*	0.148
Symptoms	4	2.3(0.98)	42%	2.5(0.96)	2.2(0.99)	8.074	0.005*	0.152
Course of disease	4	2.8(0.88)	69%	2.9(0.89)	2.7(0.87)	3.990	0.046	0.113
Assessment and Diagnosis	4	2.8(0.81)	70%	2.9(0.79)	2.7(0.84)	2.279	0.132	0.122
Treatment and Diagnosis	4	3.2(0.76)	81%	3.3(0.74)	3.2(0.80)	0.424	0.515	0.065
Life Impact	3	2.4(0.66)	81%	2.5(0.64)	2.3(0.67)	9.362	0.002*	0.151
Care Giving	5	2.1(1.12)	42%	2.2(1.13)	2.0(1.10)	1.342	0.247	0.089

*ADKS* Alzheimer’s Disease Knowledge Scale, *SD* standard deviation

*p*-value was based on Multivariate analysis of variance (MANOVA)

*ES* effect size

Bonferroni correction for multiple comparisons was applied. With 8 group comparisons conducted, a corrected *p*-value of 0.0062 was required

*Significant after Bonferroni correction (*p*-value < 0.0062)

### DCAS scores

The overall mean score of dementia attitudes was 28.5 (SD = 3.20) out of 40. Over 82% (*n* = 321) of participants thought that providing a diagnosis was usually more helpful than harmful. The majority of participants believed that much should be done to improve the quality of life of people with dementia (88.9% *n* = 347) and carers of people with dementia (81% *n* = 316). However, more than 58% (*n* = 228) of participants thought the primary care team had a limited role to play in the care of people with dementia when responding to DCAS, and only 46.6% (*n* = 182) demonstrated the positive view that ‘managing dementia was more often rewarding than frustrating’. Four demographic characteristics showed statistically significant correlations with dementia attitudes (Table 1). Females demonstrated more positive attitudes than their male counterparts (*P* = 0.031), as did participant who had experience in caring for people with dementia (*P* = 0.041). The older age of participants was associated with positive attitudes towards people with dementia (*P* = 0.045) and GPs demonstrated more positive attitudes than nurses (*P* = 0.002).

### ADCQ scores

The total mean score of the ADCQ was 7.8 (SD = 2.17). Over 85% (*n* = 332) of participants realized that it was not important to correct people with dementia. However, only 24.9 (*n* = 97) thought that people with dementia should be allowed to freely express themselves even in seemingly meaningless behavior. Participants who had experience in caring for people with dementia demonstrated more person-centered care approaches than those without experience (*P* = 0.021) (Table 1).

Moreover, professional group was not related to the dementia care approach in all content domains (Table 3).

**Table 3:** ADCQ content domains and professional group (*n* = 390)

		Professionals				
		GPs(n = 212)	Community nurses(n = 178)			
Content Domain	Mean ± SD	Mean ± SD	Mean ± SD	F value	*p*-value	ES
ADCQ (13 items)	7.8(2.17)	7.9(2.28)	7.6(2.02)	1.560	0.212	0.069
Orientation of time, place and situation (3 items)	2.1(0.75)	2.1(0.75)	2.1(0.76)	0.027	0.869	0.000
Correction of behavior (3 items)	1.5(0.84)	1.6(0.89)	1.5(0.78)	0.685	0.408	0.060
Emphasis on the past or the present (3 items)	1.7(0.95)	1.8(0.98)	1.6(0.91)	3.914	0.049	0.105
Aim of the nurses’ communication (3 items)	2.1(0.79)	2.2(0.81)	2.1(0.76)	0.480	0.489	0.064
Whether confusion had any meaning for the patient (1 item)	0.4(0.48)	0.5(0.03)	0.5(0.04)	0.351	0.554	0.000

*ADCQ* Approach to Advanced Dementia Care Questionnaire, *SD* standard deviation

*p*-value was based on Multivariate analysis of variance (MANOVA)

*ES* effect size

Bonferroni correction for multiple comparisons was applied. With 6 group comparisons conducted, a corrected *p*-value of 0.0083 was required

### Relationship between the ADKS scores, DCAS scores and ADCQ scores

A positive relationship remained between knowledge scores and attitude scores (*r* = 0.379, *P* < 0.001). A similar relationship was reported between attitude scores and care approach scores (*r* = 0.143, *P* < 0.001), while dementia knowledge had no clear relationship with the person-centred dementia care approach (*r* = 0.005, *P* = 0.921).

### Predictors for the ADKS scores, DCAS scores and ADCQ scores

Variables affecting dementia knowledge and attitudes reported in the literature and identified in the bivariate analysis in this study were entered as independent in the three separate multivariate regression models. These independent variables are age, gender, marital status, education, professional group (GPs or nurses), and experience in caring for people with dementia, separately. The results revealed that age, gender, professional group and experience in caring for people with dementia were predictors of the ADKS scores (F = 8.715 *P* = 0.000), explaining 12% of the variance in knowledge in the model (Table 4). Moreover, only gender and professional group were predictors of the DCAS scores (F = 5.598 *P* = 0.000), accounting for 8% of the total variance in dementia attitudes (Table 4). However, the multiple regression model of the ADCQ was not statistically significant (F = 1.349 *P* = 0.234).

**Table 4:** Multiple regression analysis for the ADKS and DCAS (n = 390)

	Independent variables	B	SE	β	*t* value	*p*-value
ADKS	(Constant)	17.62	0.57		31.21	0.000
	Age(years)	0.05	0.02	0.16	2.59	0.010*
	Gender(male = 1)	−1.55	0.40	−0.21	−3.88	0.000*
	Marital Status(married = 1)	−0.10	0.37	−0.02	− 0.27	0.786
	Education(diploma = 1)	−0.55	0.31	−0.09	− 1.76	0.080
	Professional group(Doctor = 1)	1.44	0.35	0.23	4.08	0.000*
	Experience in caring for people with dementia(yes = 1)	0.75	0.30	0.12	2.54	0.012*
	*R* = 0.347 R^2^ = 0.120 Adjusted R^2^ = 0.106 F = 8.715 (P = 0.000)*					
DCAS	(Constant)	27.13	0.65		42.06	0.000
	Age(years)	0.02	0.02	0.05	0.80	0.424
	Gender(male = 1)	−1.87	0.46	−2.22	−4.08	0.000*
	Marital Status(married = 1)	−0.04	0.42	−0.01	−0.09	0.926
	Education(diploma = 1)	−1.18	0.36	−0.03	−0.50	0.618
	Professional group(Doctor = 1)	1.65	0.40	0.24	4.09	0.000*
	Experience in caring for people with dementia(yes = 1)	0.78	0.34	0.11	2.31	0.021
	*R* = 0.284 R^2^ = 0.081 Adjusted R^2^ = 0.066 F = 5.598 (P = 0.000)*					

*ADKS* Alzheimer’s Disease Knowledge Scale, *DCAS* Dementia Care Attitudes Scale, *B* unstandardized coefficients, *SE* Std. error of B, *β*, standardized coefficients

*p < 0.05

## Discussion

This is the first study to assess community health professionals’ knowledge, attitudes and care approach of dementia in China. It is of fundamental importance to understand the community health professionals’ perspectives so that appropriate education interventions and policies can be designed and implemented to develop a competent dementia workforce in the community setting to respond to the increasing number of people living with dementia in China. Our findings support previous studies that older age, advanced education levels and experience in taking care of people with dementia are positively associated with health professionals’ knowledge and attitudes [24, 37]. Dementia education programme design needs to consider these factors in order to provide tailored programmes for health professionals.

This study suggests that dementia knowledge is poor among community health professionals in China. The mean knowledge scores (19.7) are lower than those reported in dementia professionals in the United States [23] and health care staff in Australia [38]. This study reveals that the lowest knowledge scores relate to dementia symptoms (Table 2). Dementia symptoms are widely considered to be part of normal ageing and not thought to be treatable, especially in community settings in China [1, 39]. Previous studies identified that lack of knowledge about the early signs and symptoms of dementia among health professionals is a barrier to achieving early detection and timely diagnosis of dementia [1, 40]. The findings of this study also reveal the lack of knowledge about caregiving among health professionals. The findings also support previous studies of Norwegian psychologists and Maltese nursing students [37, 41]. In our study, 51% of participants (*n* = 199) agreed with an incorrect statement that ‘once people have Alzheimer’s disease, they are no longer capable of making informed decisions about their own care’ (item 16 in the ADKS). This is evidence that health professionals need to know about care giving in order to enable people with dementia to maintain autonomy and independence.

Effective dementia management relies on a solid knowledge base about the pathophysiology, psychology, pharmacotherapeutics and caregiving of dementia among health professionals. Studies report that pre-registration education programmes for health professional students do not have stand-alone dementia topics in their curricula and there are limited dementia-specific continuing education programmes available for health professionals in China [42, 43]. Managing the health of people with dementia in the community requires ongoing education and specialized knowledge in dementia care for health professionals involved in primary care [44, 45]. Therefore, an audit of dementia content in pre-registration and continuing education programmes should be undertaken to ensure adequate dementia topics and content are embedded in curricula in China.

The study reveals that health professionals have generally positive attitudes despite the low levels of dementia knowledge. The findings reveal that most health professionals recognize the significance of dementia diagnosis, management and caregiving. Similar findings are reported in previous studies [12, 46]. Our study revealed that community health professionals in China demonstrate more negative attitudes toward the role of primary care teams in dementia care in their responses to the DCAS statement ‘The primary care team has a limited role to play in the care of people with dementia’ (item 7) compared to their counterparts in UK [12, 46]. This finding may indicate that community health professionals in this study tend not to regard dementia care as part of their professional duties. This is not surprising since in China currently, the main tasks of community health professionals are to provide care for older people with chronic illness limited to hypertension, diabetes and mental diseases. People with dementia but without the above conditions are not eligible to receive government subsidized services provided by community health professionals. Moreover, our study reveals that more health professionals agree that managing dementia is more often frustrating compared to those in community care settings in UK [12].

The findings of this study differ from prior studies in developed countries in that community nurses demonstrate more negative attitudes towards dementia compared with GPs. In developed countries, community nurses usually act at an advanced nursing practice level and have more autonomy in leading care services including providing care for people with dementia. For example, community mental health nurses are seen as potential specialists or advanced nurse practitioners in dementia care in the UK [47–49]. However, community nurses in Chinese community settings mainly assist GPs to deliver medical treatment and have limited autonomy in leading care services [4, 39, 50]. The less positive attitudes among community nurses in this study may be due to a combination of a restricted professional role and insufficient training related to dementia care. Therefore, to remedy this situation, we suggest that the professional roles and responsibilities of community nurses in China should be adequately defined and be expanded to an advanced level. Community nurses should have opportunities to engage in dementia education and training to prepare them to cope with challenges in dementia care considering the ageing population and the increased numbers of people living with dementia in the community setting in China.

Person-centred care has been widely recognized as the gold standard in dementia care and has been viewed as a core component of dementia care competence [9, 21]. It is evident in the literature that implementing a person-centred care approach can effectively reduce the number of agitated behaviours, the usage of antipsychotic medication and relieve additional stress for people with dementia [9, 51]. However, in this study, the majority health professionals tend not to use a person-centred approach in dementia care. This result confirms the previous studies [10, 34] and a number of factors appear to have contributed to this. First, the lack of specialized education in person-centred care means that the person-centred approach is not well known among health professionals in China. Second, there is a lack of support from organizations to enable a person-centred approach, especially in the community setting with its poor resources and low staffing levels [5, 52]. Our study also supports previous studies that experience in caring for people with dementia is positively associated with a person-centred approach [10, 53]. One possible reason may be that the longer exposure to dementia enabled health professionals to realize the person-centred approach as the most rewarding for people with dementia [10, 11]. This indicates that the care experience is a major influence affecting health professionals’ approach to people with dementia.

We identified no association between knowledge and care approach in this study. This may be because that the ADKS is not a complete assessment tool, but rather contains representative items indicating the level of general knowledge about Alzheimer’s Disease [23, 38]. Furthermore, preserving the personhood of people with dementia requires health professionals to receive specialized education in person-centred care delivery. Therefore, such basic knowledge may not ensure that health professionals demonstrate a person-centred approach in dementia care [10, 34].

### Study limitations and future research directions

First, a cross-sectional design of the present study did not allow the determination of causal relationships, only associations between knowledge, attitudes and care approach. Second, the staff population in the 6 districts may not reflect community health services centres (CHSCs) in other parts of Hunan Province or other parts of China when generalizing the findings. Third, although the present study used validated instruments, it is difficult to understand the real knowledge level of dementia by using a true and false design test. In order to collect more credible and comprehensive information about dementia knowledge and attitudes, vignettes that simulate real case studies could be used as an effective tool in future studies [49, 54]. Moreover, positive attitudes and care approach in response to dementia are usually over-reported because of their social desirability. Survey methods have their limitations when detecting actual practice, and using non-participant observation methods to collect data in care settings may address these limitations. Fourth, there was a considerable amount of variance of knowledge, attitudes and care approach which cannot be explained by the socio-demographic characteristics, experience in dementia care and education levels. The multivariate regression models need to be improved to better explain the level of knowledge, attitudes and care approach in future studies. For example, the inclusion of dementia education and training in person-centred care might be an obvious next step in attempting to develop a predictive model that explains more of the variance.

## Conclusion

This study reveals that community health professionals in China demonstrate low levels of dementia knowledge and tend not to use a person-centred approach to care for people with dementia, while having generally positive attitudes towards dementia.

Dementia is a significant public health issue in China in the context of a rapidly ageing population. Most people with dementia live in the community and are cared for by their family caregivers. Inadequate knowledge, attitudes and approaches to dementia care among health professionals in primary care are associated with negative impacts on health and quality of life for people with dementia and their family caregivers and are a burden on health care and social care systems in the country. The results indicate an urgent need for better educational preparation in dementia care. Education institutions and providers need to take action to embed dementia education into curricula for health professional students or continuing professional development programmes for those who work in the community setting. Further research to identify appropriate curriculum design, teaching and learning strategies to improve dementia knowledge, attitudes and care approach, and to translate them into practice to improve care outcomes for people with dementia is much needed.

## Abbreviations

ADCQ: Approach to Advanced Dementia Care Questionnaire
ADKS: Alzheimer’s Disease Knowledge Scale
BPSD: Behavioral and psychological symptoms of dementia
CHSCs: Community health services centres
DCAS: Dementia Care Attitude Scale
GPs: General practitioners
LMICs: Low and middle-income countries
